# Complete genome sequence of *Methanospirillum hungatei* type strain JF1

**DOI:** 10.1186/s40793-015-0124-8

**Published:** 2016-01-06

**Authors:** Robert P. Gunsalus, Lauren E. Cook, Bryan Crable, Lars Rohlin, Erin McDonald, Housna Mouttaki, Jessica R. Sieber, Nicole Poweleit, Hong Zhou, Alla L. Lapidus, Hajnalka Erzsebet Daligault, Miriam Land, Paul Gilna, Natalia Ivanova, Nikos Kyrpides, David E. Culley, Michael J. McInerney

**Affiliations:** Department of Microbiology, Immunology, and Molecular Genetics, University of California, Los Angeles, CA 90095-1489 USA; UCLA DOE Institute for Genomics and Proteomics, University of California, Los Angeles, CA 90095-1489 USA; Department of Botany and Microbiology, University of Oklahoma, Norman, OK 73019 USA; Theodosius Dobzhansky Center for Genome Bionformatics, St. Petersburg State University, St. Petersburg, Russia; Algorithmic Biology Lab, St. Petersburg Academic University, St. Petersburg, Russia; Oak Ridge National Laboratory, Oak Ridge, Tennessee USA; DOE Joint Genome Institute, Walnut Creek, CA USA; Department of Biological Sciences, King Abdulaziz University, Jeddah, Saudi Arabia; Pacific Northwest National Laboratory, Richland, WA USA

**Keywords:** *Methanomicrobiales*, Anaerobic, Motile, Methangenic archaea, Hydrogen, Formate, Syntrophic partnerships

## Abstract

*Methanospirillum hungatei* strain JF1 (DSM 864) is a methane-producing archaeon and is the type species of the genus *Methanospirillum*, which belongs to the family *Methanospirillaceae* within the order *Methanomicrobiales*. Its genome was selected for sequencing due to its ability to utilize hydrogen and carbon dioxide and/or formate as a sole source of energy. Ecologically, *M. hungatei* functions as the hydrogen- and/or formate-using partner with many species of syntrophic bacteria. Its morphology is distinct from other methanogens with the ability to form long chains of cells (up to 100 μm in length), which are enclosed within a sheath-like structure, and terminal cells with polar flagella. The genome of *M. hungatei* strain JF1 is the first completely sequenced genome of the family *Methanospirillaceae*, and it has a circular genome of 3,544,738 bp containing 3,239 protein coding and 68 RNA genes. The large genome of *M. hungatei* JF1 suggests the presence of unrecognized biochemical/physiological properties that likely extend to the other *Methanospirillaceae* and include the ability to form the unusual sheath-like structure and to successfully interact with syntrophic bacteria.

**Electronic supplementary material:**

The online version of this article (doi:10.1186/s40793-015-0124-8) contains supplementary material, which is available to authorized users.

## Introduction

Strain JF1 (DSM 864 = ATCC 2790D-5) [1] is the type species for *M. hungatei* and represents the first isolated member of the *Methanospirillaceae* within the order *Methanomicrobiales* [2]. The species epithet derives from the Latin and honors Dr. R. E. Hungate, the inventor of methodologies for modern isolation and cultivation of strictly anaerobic bacteria and archaea [3, 4]. *M. hungatei* strain JF1 was isolated from a secondary anaerobic sewage treatment digestor in Urbana, Illinois, as part of a study of anaerobic aromatic hydrocarbon metabolism [5].

Here, we describe the genome sequence of *M. hungatei* strain JF1, a hydrogen- and formate-utilizing, methane-producing archaean. The genomic data provide insight towards defining the unique genes needed for anaerobic syntrophy [6], which occurs within a phylogenetically diverse range of bacteria, and for classifying genes identified by environmental DNA sequencing projects.

## Organism information

### Morphology and physiology

**Fig. 1:**
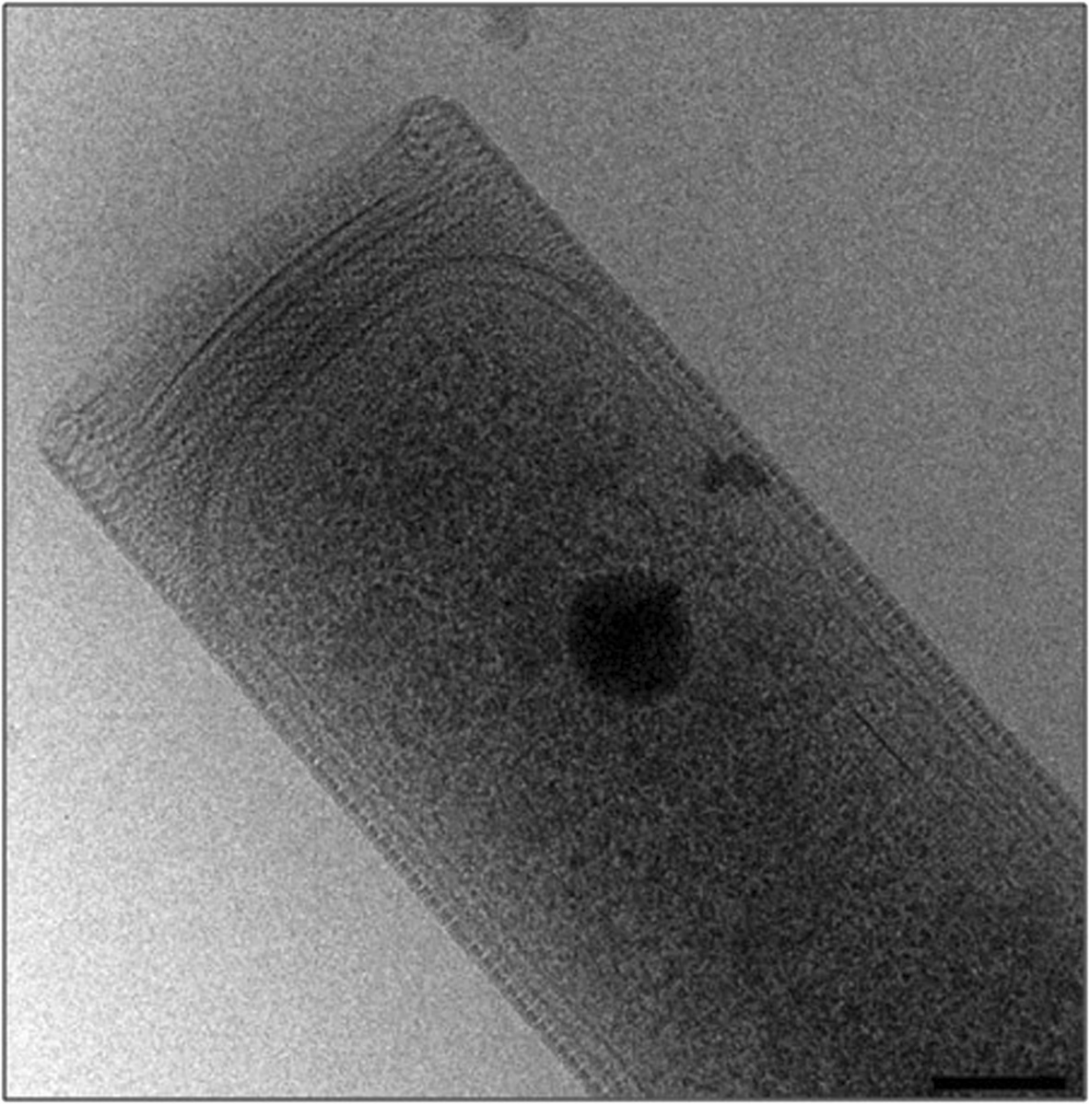
Electron micrograph of *M. hungatei* strain JF1 cells and associated sheath structure. Scale bar corresponds to 100 nm

Cells of *Methanospirillum hungatei* strain JF1 are narrow, curved rods (i.e., spirillum shaped) that measure ~0.5 μm by ~7 μm in size (Fig. 1, Table 1). The cells are contained within a sheath-like structure that contain one or more cells; the sheath may extend to over 100 μm in length depending on the nutritional conditions [1, 7]. Individual cells stain Gram-negative and are weakly motile by polar tufts of flagella. Cells also possess polyphosphate bodies or granules located at opposing cell ends [8]. Growth and metabolism is strictly anaerobic where hydrogen plus carbon dioxide and/or formate serve as the methanogenic substrate. Acetate is required as the major supply for cell carbon [1, 7]. Cells have no other organic nutritional requirements although addition of Casamino Acids or other plant/animal hydrolysis products speeds growth [1]. Temperature range for growth is 20–40 °C (optimum at 37 °C).

**Table 1:** Classification and features of *Methanospirillum hungatei* strain JF1 according to MIGS recommendations [45] published by the genomic standards consortium [46] and the names for life database [47]

MIGS ID	Property	Term	Evidence code^a^
	Current classification	Domain *Archaea*	TAS [48]
		Phylum *Euryarchaeota*	TAS [49]
		Class *Methanomicrobia*	TAS [50]
		Order *Methanomicrobiales*	TAS [51]
		Family *Methanospirillaceae*	TAS [2]
		Genus *Methanospirillum*	TAS [1]
		Species *Methanospirillum hungatei*	TAS [1]
		Type strain JF-1	TAS [1]
	Gram stain	Negative	TAS [1]
	Cell shape	Curved rods 0.5 μM x 7.4 μM	TAS [1]
	Motility	Motile	TAS [1]
	Sporulation	Non-sporulating	TAS [1]
	Temperature range	30 °C-40 °C	TAS [1]
	Optimum temperature	37 °C	TAS [1]
	pH range; Optimum	6.5–10; 6.6–7.4	TAS [2]
	Carbon source	Carbon dioxide, formate, acetate	TAS [1]
	Energy source	Hydrogen, formate	TAS [1]
	Terminal electron receptor	Carbon dioxide	TAS [1]
MIGS-6	Habitat	Anaerobic sediments, sewage digesters	TAS [1]
MIGS-6.3	Salinity	Fresh to brackish water	TAS [1]
MIGS-22	Oxygen requirement	Strict anaerobe	TAS [1]
MIGS-15	Biotic relationship	Syntrophic	TAS [1]
MIGS-14	Pathogenicity	Non-pathogen	TAS [1]
MIGS-4	Geographic location	USA, Urbana, IL	TAS [1]
MIGS-5	Sample collection time	1972	TAS [1]
MIGS-4.1	Latitude	40.109°N	NAS
MIGS-4.2	Longitude	88.204°W	NAS
MIGS-4.4	Altitude	222 m	TAS [1]

These evidence codes are from the Gene Ontology project [52]

*IDA* Inferred from Direct Assay, *TAS* Traceable Author Statement (i.e., a direct report exists in the literature); *NAS* Non-traceable Author Statement (i.e., not directly observed for the living, isolated sample, but based on a generally accepted property for the species, or anecdotal evidence)

^a^Evidence codes

Biogenic methane production is important in the global carbon cycle and is used to treat sewage and other organic wastes and to produce biofuel from biomass [9, 10]. The degradation of fatty and aromatic acids is often the rate-limiting step in methanogenesis [6]. Fatty and aromatic acid degradation is thermodynamically favorable only when hydrogenotrophic methanogens such as *M. hungatei* strain JF1 maintain very low levels of hydrogen and/or formate in a process called syntrophy [10, 11]. Members of the genus *Methanospirillum* are often detected in ecosystems where syntrophy is essential [1, 12] and *M. hungatei* strain JF1 is the model partner in syntrophic cocultures of the propionate degrader *Syntrophobacter wolinii* [13], the butyrate degrader *Syntrophomonas wolfei* [14], and the benzoate degraders *Syntrophus buswellii* and *Syntrophus aciditrophicus* [15, 16].

### Classification and features

**Fig. 2:**
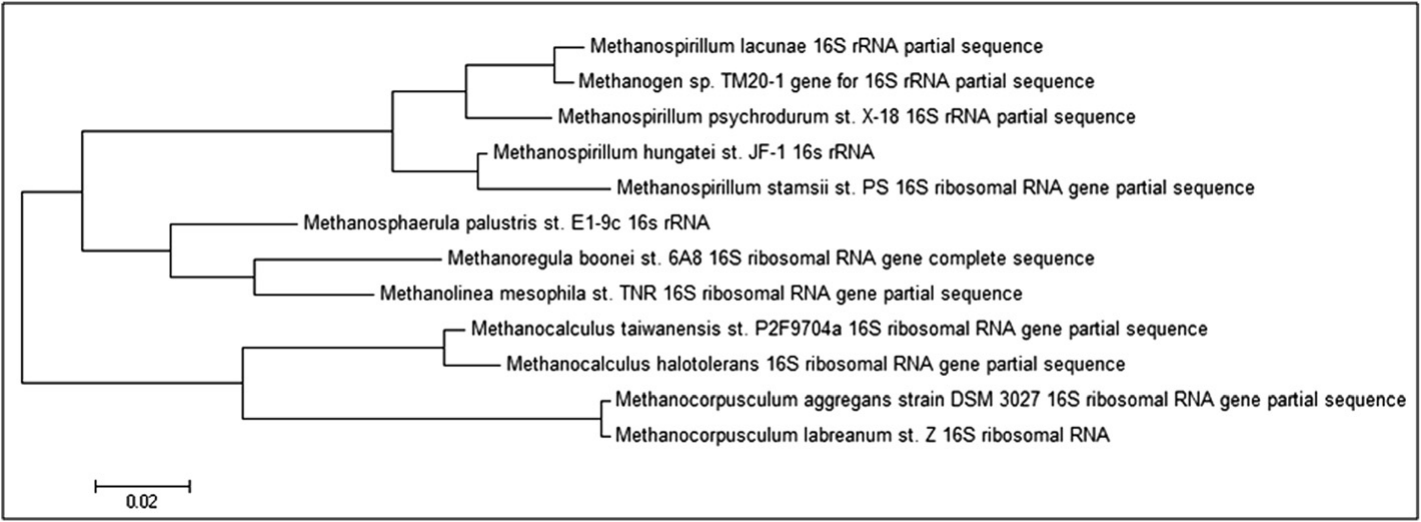
Phylogenetic tree highlighting the position of *Methanosprillulm hungatei* strain JF1 relative to other type strains within the *Methanomicrobiales.* The evolutionary history was inferred by using the Maximum Likelihood method based on the Tamura-Nei model [43]. The tree with the highest log likelihood (−3033.8513) is shown. Initial tree(s) for the heuristic search were obtained automatically by applying Neighbor-Join and BioNJ algorithms to a matrix of pairwise distances estimated using the Maximum Composite Likelihood (MCL) approach, and then selecting the topology with superior log likelihood value. The tree is drawn to scale, with branch lengths measured in the number of substitutions per site. The analysis involved 12 nucleotide sequences. Codon positions included were 1st + 2nd + 3rd + Noncoding. All positions containing gaps and missing data were eliminated. There were a total of 789 positions in the final dataset. Evolutionary analyses were conducted in MEGA6 [44]

The phylogenetic neighborhood of *M. hungatei* strain JF1 is shown in Fig. 2 for representative archaeal 16S rRNA sequences belonging to the order *Methanomicrobiales*. The four described *Methanospirillum* species form a well-defined cluster distinct from the other genera within the order where *Methanospirillum lacunae* and *Methanospirillum psychrodurum* form one subgroup and *M. hungatei* plus *Methanospirillum stamsii* form another. All strains of the genus *Methanospirillum* synthesize methane from hydrogen and carbon dioxide, though the ability to use formate is variable. None are able to ferment or respire by using other electron acceptors (i.e., with sulfate, nitrate, or iron). Certain species of other genera within the *Methanomicrobiales* also use formate, and some are reported to also metabolize short chain alcohols.

The analysis of the four 16S rRNA genes present in the *M. hungatei* JF1 genome reveled nearly identical nucleotide sequences but they differ from one another at two positions (nucleotide positions 937 and 1382) across the 1466 nucleotide length. The previously- published 16S rRNA gene sequences (AY196683 and AB517987) used in phylogenetic investigations were incomplete*,* i.e.*,* 1271 and 1259 nucleotides, respectively [17, 18].

#### Chemotaxonomic data

The cell envelope of this Gram-negative cell wall type includes a surface layer coat, also known as a surface layer protein, which surrounds the cytoplasmic membrane, and an outermost sheath structure that encapsulates multiple cells, which are arranged in chains up to 0.1 mm in length [1, 8, 19]. Cytoplasmic membrane lipids are composed primarily of biphytanyldiglycerol tetraether glycolipids [20]. *M. hungatei* strain JF1 lacks *b*-or *c*-type hemes, quinones, and methanophenazine (this study). The DNA G + C content was previously reported with 45 mol % [1].

## Genome sequencing information

### Genome project history

The *M. hungatei* strain JF1 genome was selected by DOE in 2004 as JGI sequencing project 364479 based on its phylogenetic position, its role in anaerobic decomposition of organic matter, and its ability to grow in co-culture with many syntrophic bacterial species [6]. The genome project is deposited in the Genomes OnLine Database [21] as project Id:Gc00350, and the complete genome sequence is deposited in GenBank. Sequencing, finishing, and annotation of the *M. hungatei* genome were performed by the DOE Joint Genome Institute [22]. A summary of the project information is shown in Table 2.

**Table 2:** Project information

MIGS ID	Property	Term
MIGS 31	Finishing quality	Finished
MIGS-28	Libraries used	3, 8, 14 kb
MIGS 29	Sequencing platforms	Sanger
MIGS 31.2	Fold coverage	14.5X
MIGS 30	Assemblers	PGA
MIGS 32	Gene calling method	Prodical GenePRIMP
	Locus Tag	Mhun_0000
	Genbank ID	CP000254
	GenBank Date of Release	March 1, 2006
	GOLD ID	Gc00350
	BIOPROJECT	PRJNA13015
MIGS 13	Source Material Identifier	DSM 864 T
	Project relevance	Carbon cycle, energy production, bioreactors

### Growth conditions and genomic DNA preparation

*M. hungatei* strain JF1 was grown in basal medium under anaerobic conditions at 37 ° C as previously described [1]. High molecular weight genomic DNA was isolated from cell pellets (DSM 864 = ATCC 2790D-5) using the CTAB method described at the JGI’s web site [22].

### Genome sequencing and assembly

The genome was sequenced at the Joint Genome Institute using a combination of 3 kb, 8 kb, and 40 kb DNA libraries. All general aspects of library construction and sequencing performed are described at the JGI’s web site [22]. The Phred/Phrap/Consed software package [23] was used to assemble all three libraries and to assess quality [24, 25]. Possible miss-assemblies were corrected and gaps between contigs were closed by editing in Consed, custom primer walks, or PCR amplification (Roche Applied Science, Indianapolis, IN). The error rate of completed genome sequence of *M. hungatei* is less than 1 in 50,000. The sequence of *M. hungatei* can be accessed using the GenBank accession number CP000254.

### Genome annotation

Genes were identified using Prodical [26] as part of the Oak Ridge National Laboratory genome annotations pipeline, followed by a round of manual curation using the JGI GenePRIMP pipeline [27, 28]. The predicted CDSs were translated and used to search the National Center for Biotechnology Information nonredundant database, and the UniProt, TIGRFam, Pfam, PRIAM, KEGG, COG, and InterPro databases. Additional gene prediction analysis and functional annotation was preformed within the Integrated Microbial Genomes-Expert Review platform [29, 30]. Membrane transport protein analysis was done by IMG with additional analysis by TransportDB [31] TCDB [32] databases. Transcription factor analysis and prediction was by assisted by TBD database [33].

## Genome properties

**Fig. 3:**
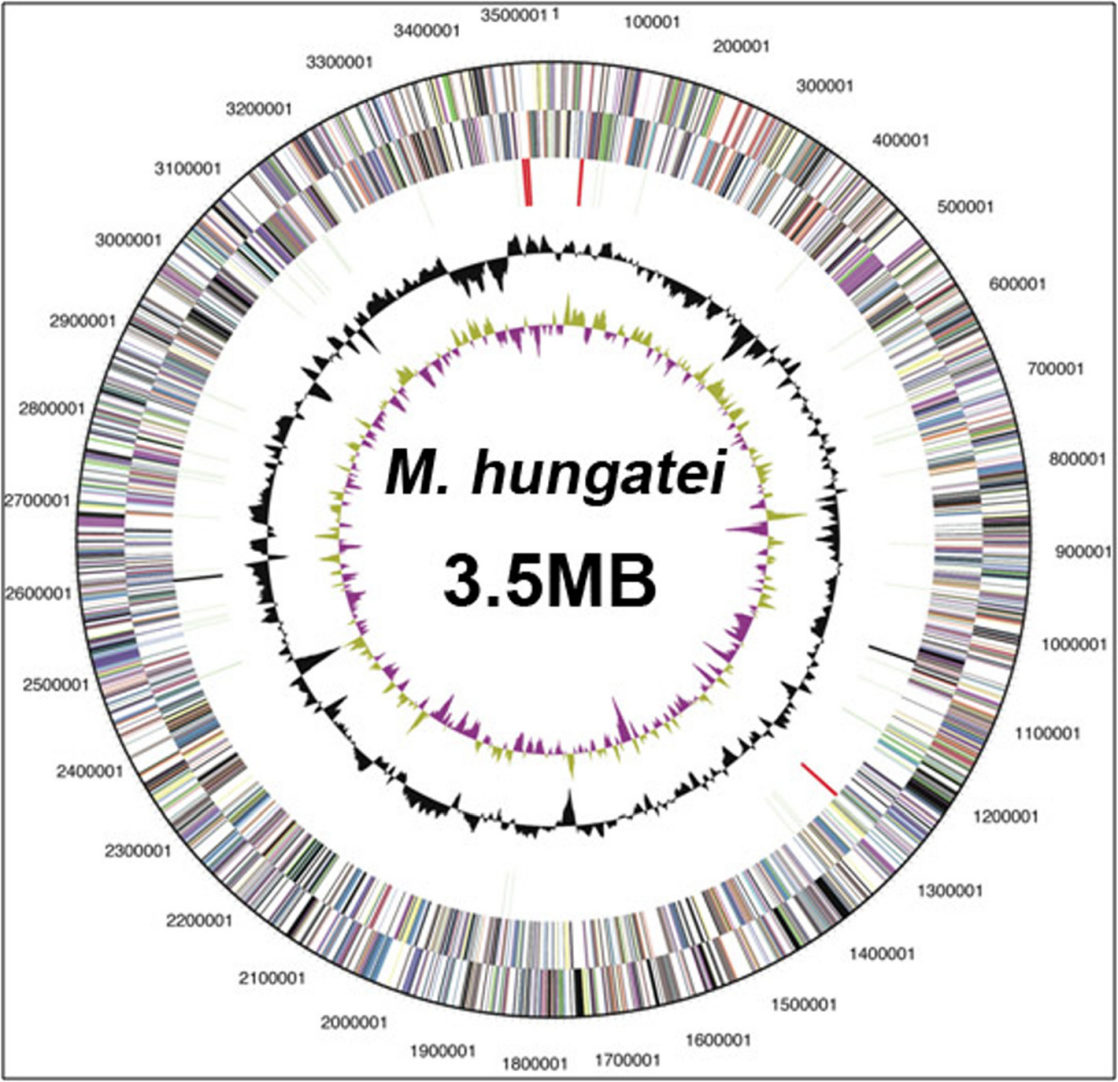
Graphic circular map of the *M. hungatei* JF1 chromosome. The concentric circles from outside to inside indicate: genes on the forward strand, genes on the reverse strand, RNA genes (tRNA’s green, .rRNA’s red, other RNA’s black), GC content, and GC skew

The genome statistics are provided in Table 3 and Fig. 3. The genome consists of one circular chromosome of 3,544,738 bp with 3,307 predicted genes of which 3,239 are protein-coding genes. Of these, approximately 61 % (2,018 genes) were assigned to a putative function while the remaining 37 % (1,221 genes) are without assigned functions. The genome is 45.15 G + C and 88.64 % coding. The distribution of genes into COGs functional categories is presented in Table 4. Of note, six CRISPER repeats were identified on the chromosome. The *M. hungatei* genome has 51 tRNA genes; 43 have identified functions, which cover all amino acids except His. The genes for histidine biosynthesis from pyruvate are present with the exception that a gene for histidinol phosphate phosphatase (HisN) was not detected. Nutritional studies [1, 7] did not detect histidine auxotrophy, suggesting that *M. hungatei* has undescribed mechanisms for fulfilling the role of HisN and synthesizing His-tRNA.

**Table 3:** Genome statistics

Attribute	Value	% of total
Genome size (bp)	3,544,738	100.00
DNA coding (bp)	3,142,074	88.94
DNA G + C (bp)	1,600,415	45.15
DNA scaffolds	1	100.00
Total genes	3,307	100.00
Protein coding genes	3,239	97.94
RNA genes	68	2.06
Pseudo genes	99	2.99
Genes in internal clusters	2172	65.68
Genes with function prediction	2,018	61.02
Genes assigned to COGs	1872	56.61
Genes with Pfam domains	2577	77.93
Genes with signal peptides	101	3.05
Genes with transmembrane helices	762	23.04
CRISPR repeats	6

**Table 4:** Number of genes associated with general COG functional categories

Code	Value	% age	Description
J	180	8.65	Translation, ribosomal structure and biogenesis
A		0.00	RNA processing and modification
K	84	4.03	Transcription
L	82	3.94	Replication, recombination and repair
B	8	0.38	Chromatin structure and dynamics
D	16	0.77	Cell cycle control, Cell division, chromosome partitioning
V	53	2.55	Defense mechanisms
T	154	7.4	Signal transduction mechanisms
M	85	4.08	Cell wall/membrane biogenesis
N	54	2.59	Cell motility
U	17	0.82	Intracellular trafficking and secretion
O	89	4.27	Posttranslational modification, protein turnover, chaperones
C	186	8.93	Energy production and conversion
G	59	2.83	Carbohydrate transport and metabolism
E	165	7.93	Amino acid transport and metabolism
F	62	2.98	Nucleotide transport and metabolism
H	162	7.78	Coenzyme transport and metabolism
I	31	1.49	Lipid transport and metabolism
P	147	7.06	Inorganic ion transport and metabolism
Q	16	0.77	Secondary metabolites biosynthesis, transport and catabolism
R	217	10.42	General function prediction only
S	160	7.68	Function unknown
-	1435	43.39	Not in COGs

The total is based on the total number of protein coding genes in the genome

## Insights from the genome sequence

### Methanogenesis pathway

**Fig. 4:**
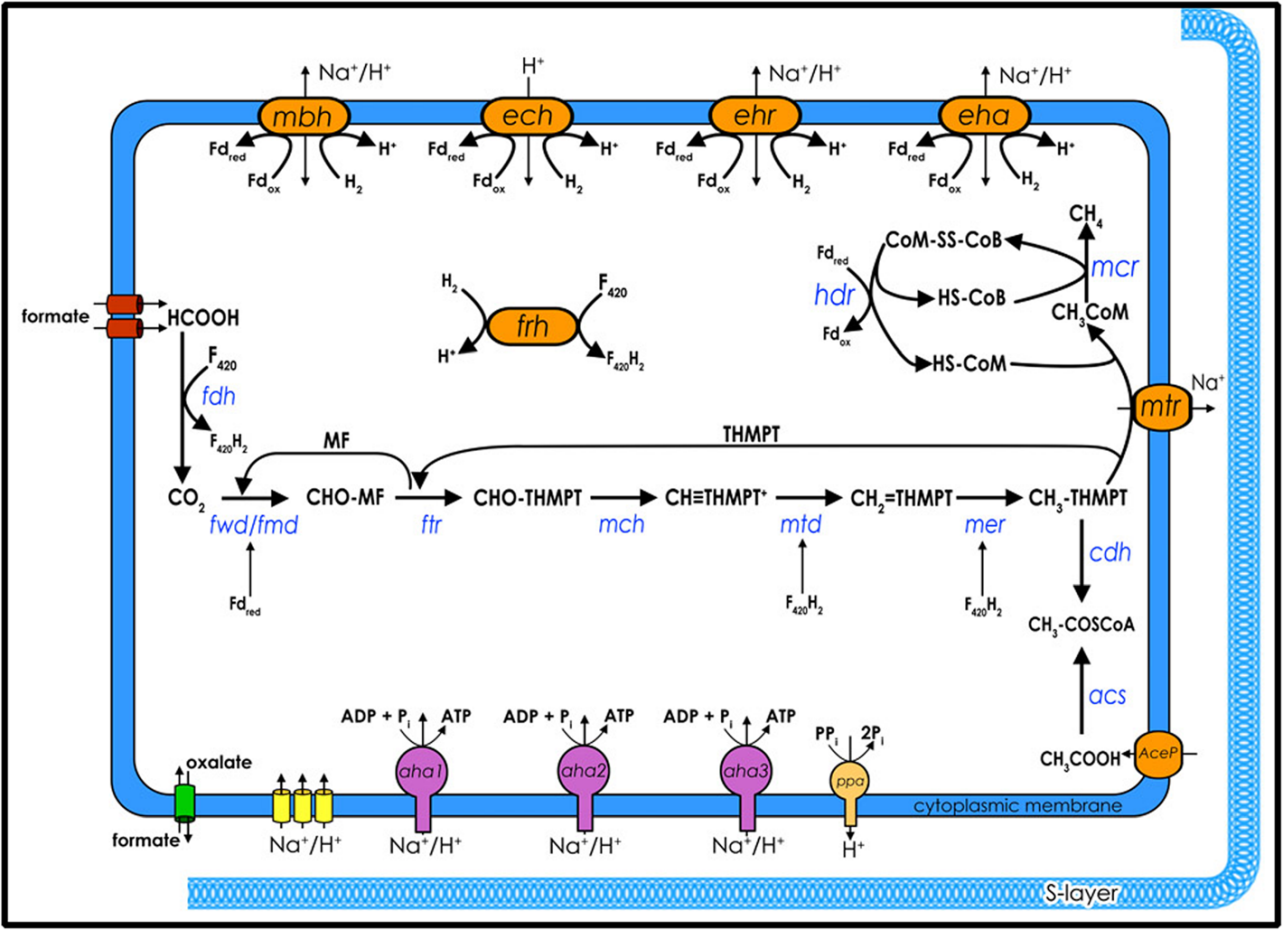
Overview of central metabolism in *M. hungatei* strain JF1. The pathway for methane formation from hydrogen and formate is shown in black with key steps shown with gene/enzyme designations. Membrane proteins involved in energy transduction electron transport, and ion/solute translocation are arranged along the cytoplasmic membrane: archaeal ATP synthase, Aha; formate dehydrogenases, Fdh; hydrogenases (Mbh, Ech, Ehr, Eha Frh); formyl-methanofuran dehydrogenase, Fmd, Fwd; methenyl–H_4_MPT tetrahydromethanopterin cyclohydrolyase, Mch; formylMFR:tetrahydromethanopterin formyl transferase, Ftr; methylene–H_4_MPT dehydrogenase, Mtd; methylene–H_4_MPT reductase, Mer; H_4_MPT S-methyltransferase (Mtr; methyl-CoM reductasem Mcr; and CoM-S-S-CoB heterodisulfide reductase, Hdr

The *M. hungatei* JF1 ORFs were organized into pathways where most pathways considered essential for viability of a typical archaeal cell were detected. The methanogenic pathway from hydrogen and carbon dioxide is highly conserved in methanogens and the genes for all the enzymes in the central methanogenic pathway were identified, including a soluble-type heterodisulfide reductase only (Fig. 4). The genome contains three gene sets for molybdenum (*fmd*) or tungsten (*fwd*) type formylmethanofuran (MFR) dehydrogenases (Mhun_1981-84, Mhun_1985-94 and Mhun_210612) that catalyze the ferredoxin-dependent first step of carbon dioxide reduction. There are three genes for methenyl–H_4_MPT tetrahydromethanopterin (H_4_MPT) cyclohydrolyase (Mch: Mhun_0022, Mhun_0444, Mhun _2384), which catalyze the third pathway step.

Single genes encode enzymes for the second, fourth, and fifth pathway steps, formylMFR:tetrahydromethanopterin formyl transferase (Ftr: Mhun_1808), methylene–H_4_MPT dehydrogenase (Mtd: Mhun_2255) and methylene –H_4_MPT reductase (Mer: Mhun_2257). The latter two enzymes employ reduced cofactor F_420_ as substrate. The remaining two enzymes in the pathway are multi-subunit complexes: H_4_MPT Smethyltransferase (Mtr: Mhun_2168-75), and the type I methyl-CoM reductase (Mcr: Mhun_2144-2148). The CoM-S-S-CoB heterodisulfide reductase (Hdr: Mhun_1834-39) so named for the methanogenic co-enzymes M and B, reduces CoM-S-S-CoB hertodisulfide generated by Mcr. The reaction catalyzed by a soluble-type Hdr is likely an electron bifurcation, which couples the energetically favorable reduction of CoM-S-SCoB by formate and/or H_2_ with the energetically unfavorable reduction of ferredoxin by formate and/or H_2_ [34].

The oxidation of hydrogen or formate is needed to generate reduced ferredoxin and cofactor F_420_ used in several of the above reactions (Fig. 4). The oxidation of hydrogen or formate may be accomplished by one or more of the multiple hydrogenase and formate dehydrogenase enzymes. Five nearly identical gene clusters encode soluble formate dehydrogenase (Fdh) enzymes: Mhun_1813-1814, Mhun_1832-1833, Mhun_2020-2021, Mhun_2022-2023, and Mhun_3237-3238. There are two formate/nitrite-type transporters (Mhun_0075, Mhun_1811). The five hydrogenase gene clusters include *echABDDEF* (Mhun_1741-1747), *ehrABCDLS* (Mhun_1817-1822), *ehaABCDEFGHIJK* (Mhun_2094-2106), *frhADGB* (Mhun_2329-2332), and *mbhABCDEFGHIJKLMN* (Mhun_2579-2592). The *ech, eha, ehr*, and *mbh* gene clusters encode membrane-associated enzymes that likely reduce Fd. These are believed to employ ion gradients (Na^+^ or H^+^) to assist Fd reduction at low hydrogen levels. The remaining hydrogenase gene cluster (*frh*ADGB) encodes a soluble hydrogenase that reduces F_420_.

### Transporters, ion movement, and ATP synthesis

*M. hungatei* JF1 has 352 genes involved in membrane transport as determined by IMG/ER, which constitute 10.64 % of the genome. These include 34 multi-component ATP-binding cassette or ABC-type transporter genes plus related but unlinked genes (152 genes in total), sixty genes encoding secondary transporters, twelve genes for ion channels, seven genes for P-ATPases, one H^+^ translocating pyrophosphatase (Mvp, H + PPase; Mhun_2414) gene, and four type II secretion systems. A highly unusual feature of the *M. hungatei* genome is the presence of three H^+^ or Na^+^ -translocating AoA_1_-type ATP synthetase gene clusters encoded by 27 genes (Aha1, Mhun_1177-1185; Aha2, Mhun_1757-1765, and Aha3, Mhun_1768-1775). The gene order is conserved relative to the corresponding Aha complex in *Methanosarcina acetivorans* [35]. Although it is unknown whether these systems utilize protons or sodium ions, the *M. acetivorans* ortholog is believed to use sodium ions [35]. Likewise, the membrane-bound H_4_MPT Smethyltransferase (Mtr) is predicted to be sodium dependent. Three genes encode Na^+/^H^+^ antiporters (Mhun_0680, Mhun_0841, Mhun_2803) that might maintain ion balance where the last differs by also possessing a Trk domain.

### Cell biosynthesis

The genome of *M. hungatei* encodes an acetyl-CoA synthase/CO dehydrogenase complex (Cdh; Mhun_0686-0690). The role of Cdh is undefined at this time because *M. hungatei* must acquire acetate supplied in the medium for growth rather than synthesizing acetylCoA from CO_2_, which is the usual role of Cdh in hydrogenotrophic methanogens. Uptake of acetate for incorporation into cell material is predicted to occur by the Mhun_0634 *aceP* gene product [35]. Five acetyl-CoA synthetase genes are present that could activate acetate to acetyl-CoA. Mhun_0352, Mhun_0567, and Mhun_1721 share > 62 % identity at the amino acid level with each other, but only share < 34.2 % amino acid identity with Mhun_0592 and Mhun_2392.

*M. hungatei* has two set of genes that could be used to carboxylate acetyl-CoA to pyruvate (Mhun_2393-2396 and Mhun_0450-0453). Oxaloacetate can be synthesized by carboxylation of pyruvate using pyruvate carboxylase (Mhun_3189-3190) or by conversion of pyruvate to phosphoenol pyruvate by pyruvate dikinase (Mhun_2610 or Mhun_1141) and carboxylation of phosphoenol pyruvate to oxaloacetate by phosphoenol pyruvate carboxylase (Mhun_0174). The genes necessary to convert oxaloacetate to 2-oxoglutarate by the reductive arm of the tricarboxylic acid cycle were detected (malate dehydrogenase, Mhun_1155; fumarate hydratase, Mhun_0089-0090; succinyl-CoA ligase, Mhun_0096-0095; 2-oxoglutarate synthase, Mhun_0091-0094 or Mhun_29922994; and fumarate reductase, Mhun_3052-3053). Complete biosynthetic pathways for the synthesis of all amino acids except histidine from pyruvate, oxaloacetate, and 2-oxoglutarate as the main starting materials were detected.

There are few genomic clues regarding the composition of the *M. hungatei* cell envelope. The genome contains a large number of PDK domain-containing genes (31 genes) as well as TRP domain-containing genes (41 genes). Many of these have transmembrane and/or SP signal elements that would suggest cell envelope associations but it is unknown if any of the proteins are significantly expressed. There are no clear protein candidates for the morphologically defined cell envelope structures containing a surface layer, sheath, and plugs [1, 8].

### Stress

There appear to be few cellular adaptations in *M. hungatei* for stress response. Among those found are defense against oxygen damage: catalase (Mhun_2433), peroxidase (Mhun_2733), manganese/iron superoxide dismutase (Mhun_2974), heavy metal resistance (Mhun_1348, Mhun_3034), drug resistance (Mun_0598, Mhun_1195) and heat shock (Mhun_2436).

### Regulation and signal transduction

The *M. hungatei* genome contains a typical set of archaeal RNA polymerase genes and one BRE recognition factor analogous to eukaryotic transcription initiating factor B (Mhun_2481; Tfb) plus two TATA-box binding proteins or TBP’s that confer promoter recruitment and specificity (Tbp1, Mhun_0568 and Tbp2, Mhun_0593). There are ~65 DNA-binding transcription factors identified that modulate gene expression. These belong to a variety of protein families common to bacteria but include few regulatory proteins typical of eukaryotes (e.g., homeodomain-like, zinc finger, SRF-like, or p53-like proteins). There are numerous bacterial-type two-component regulatory systems including 82 histidine kinase-type sensor transmitters, 41 response regulatory proteins, and 18 receiver-only domain proteins. Of the 82 histidine kinases, 55 are soluble and 27 are membrane-associated. They are generally unlinked genetically and thus do not suggest an interacting partner in sensory transduction.

### Motility and taxis

*M. hungatei* JF1 possesses multiple archaeal-type flagella filaments at the cell ends [1, 8], now termed archaealla that resemble bacterial type IV pili [36, 37]. The genome contains one *flhGFHIJ* gene cluster (Mhun_0102-0105) encoding a basal body structure. Three FlaB-type pili genes make up the archaella filaments (Mhun_1238, Mhun_3139,

Mhun_3140). Although little is known about the chemotactic abilities of *M. hungatei*, other than movement towards an essential nutrient, acetate [38], there are multiple chemosensory genes present in the genome. These include 3 CheA, 4 CheB, 4 CheC, 1 CheD, 3 CheR, 1 CheY, and 14 CheW, genes plus 27 genes encoding MCP sensory proteins (methyl accepting chemotaxis proteins) that detect unknown attractants and/or repellants. Twelve MCPs are membrane-associated and 15 MCPs are soluble.

Multiple genes (~11 paralogs) are also present in the *M. hungatei* JF1 genome for archaeal-type pili like those seen in *Methanococcus maripaludis**,**Haloferax volcanii*, and *Sulfolobus acidocaldarius* [39]. These archaeal proteins, distinct from the bacterial pili-type proteins, were previously annotated as hypothetical genes (e.g., Mhun_0297). The *H. volcanii* pili proteins provide adhesion to surfaces and the orthologs in *M. hungatei* JF1 may function in cell-cell adhesion or in cell-cell communication, although such appendages have not been previously observed in EM micrographs. All but one of the eleven *M. hungatei* JF1 paralogs are in clusters of 2 to 3 genes each and often with ABCtype transport genes.

### Comparison to other archaeal genomes

The 3.54 MB *M. hungatei* JF1 genome is the largest within the order *Methanomicrobiales* that have been sequenced thus far including *Methanosphaerula palustris* (2.92 MB) and *Methanocorpusculum labreanum* (1.80 MB). The *M. hungatei* JF1 genome is also among the largest within the *Archaea* domain: only three species sequenced thus far, belonging to the genus *Methanosarcina* (i.e., *Methanosarcina acetivorans*, 5.75 MB; *Methanosarcina barkeri*, 4.87 MB; and *Methanosarcina mazei*, 3.83 MB), plus one halophile, *Haloarcula marismortui* (4.27 MB), exceed it in size. The large genome of *M. hungatei* JF1 suggests the presence of unrecognized biochemical/physiological properties that likely extend to the other *Methanospirillaceae* and include the ability to form the unusual sheath-like structure and to successfully interact with syntrophic bacteria.

When *M. hungatei* ORFs were compared pair-wise to individual microbial genomes [40, 41], best reciprocal BLAST hits revealed closest associations to the taxonomically related archaea: *Methanoculleus marisnigri* (1395 reciprocal gene hits), *Methanosarcina acetivorans* (1203), and *Methanosarcina barkeri* (1150), and extending to *Haloquadratum walsbyi* (657) (Additional file 1: Figure S1). Thus, approximately 650 to 1,200 genes are similar and well-conserved across these 17 archaeal species whereby the remaining genes (ca. 1700 genes) represent a novel complement within the *M. hungatei* genome. Interestingly, seven of the next thirteen closest matches are bacterial species among which are many syntrophic microorganisms that likely grow in close association with *M. hungatei*. Strikingly, *Syntrophobacter fumaroxidans* strain MPOB exhibited 634 best reciprocal BLAST hits.

In another comparison, the best BLAST hit to any microbial gene product was determined (Additional file 2: Figure S2) and showed 1; 167; 277; and 142 ORFs closest hits in the genomes of *Methanoculleus marisnigri**,**Methanocorpusculum labreanum**, and**Methanosarcina barkeri*, respectively. Notably three bacterial genomes, *Syntrophus aciditrophicus**,**Syntrophobacter fumaroxidans*, and *Nostoc**spp.* gave 21–19 best BLAST hits each, suggesting the possibility of lateral gene transfer events from these potential syntrophic partners. The occurrence of *Nostoc*-related genome sequences raises interesting questions concerning microbial interactions and lateral gene transfer with methanogens present in complex microbial communities [42].

### Extended insights

The large genome of *M. hungatei* JF1 suggests the presence of unrecognized biochemical/physiological properties that likely extend to the other *Methanospirillaceae* and include the ability to form the unusual sheath-like structure and the ability to successfully interact with syntrophic bacteria. A number of genes may have been acquired by lateral gene transfer from its syntrophic partners or other microorganisms present in complex microbial communities. Also of particular note are multiple genes for archaeal type IV pili that may function in cell-cell adhesion or cell-cell communication and genes for multiple hydrogenases and formate dehydrogenases to metabolize hydrogen and formate generated by its syntrophic partners. The core machinery of *M. hungatei* to produce methane from hydrogen and carbon dioxide and/or formate is typical of other hydrogenotrophic methanogens, except that *M. hungatei* has genes for three H^+^ or Na^+^-translocationg A_O_A_1_-type ATP synthases. *M. hungatei* has four 16S ribosomal RNA genes that each differ at two positions. Further understanding of the novel compliment of *M. hungatei* genes will likely provide a more thorough understanding of the multispecies interactions involved in syntrophy and the synthesis of complex structures such as the *M. hungatei* sheath, which is shared by multiple cells.

## Conclusions

We report here an inventory of the genomic features of the methane-producing anaerobic archaeon, *Methanospirillum hungatei* strain JF1 (DSM 864), and describe its phylogenetic relationship to its neighbors. We further identify from the sizable genome of *M. hungatei* examples of genes involved in anaerobic syntrophy, and as the type strain of the *Methanospirillum*, suggest potential universal qualities of this genus. We hope this report aids and stimulates further study of this fascinating organism.

## Additional files

Additional file 1: Figure S1. Best reciprocal protein hits for *M. hungatei* JF1 ORFs with other genomes. (DOCX 89 kb) Additional file 2: Figure S2. Best Blast hit distribution of *M. hungatei* JF1 ORFs with other genomes. (DOCX 62 kb)

## References

[1] Ferry JG, Smith PH, Wolfe RS (1974). *Methanospirrillum*, a new genus of methanogenic bacteria. Int J of Syst Bacteriol.

[2] Boone DR, Whitman WB, Koga Y, Boone DR, Castenholz RW (2001). Family III. *Methanospirillaceae* fam. nov. Bergey’s Manual of Systematic Bacteriology.

[3] Hungate RE (1950). The anaerobic mesophilic cellulolytic bacteria. Bacteriol Rev.

[4] Wolfe RS (2011). Techniques for cultivating methanogens. Methods in Ezymology.

[5] Ferry JG, Wolfe RS (1976). Anaerobic degradation of benzoate to methane by a microbial consortium. Arch Microbiol.

[6] McInerney MJ, Struchtemeyer CG, Sieber J, Mouttaki H, Stams AJM, Schink B (2008). Physiology, ecology, phylogeny, and genomics of microorganisms capable of syntrophic metabolism. In, Incredible Anaerobes: From Physiology to Genomics to Fuels. Edited by Wiegel J, Maier R, and Adams M. Anal NY Acad Sci.

[7] Ferry JG, Wolfe RS (1977). Nutritional and biochemical characterization of *Methanospirillum hungatii*. Appl Environ Microbiol.

[8] Toso DB, Henstra A-M, Gunsalus RP, Zhou ZH (2011). Structural, mass, and elemental analyses of storage granules in methanogenic archaeal cells. Environ Microbiol.

[9] McInerney MJ, Sieber JR, Gunsalus RP (2009). Syntrophy in anaerobic global carbon cycles. Curr Opin Biotechnol.

[10] Schink B (1997). Energetics of syntrophic cooperation in methanogenic degradation. Microbiol Mol Biol Rev.

[11] Sieber JR, McInerney MJ, Gunsalus RP (2012). Genomic insights into syntrophy: the paradigm for anaerobic metabolic cooperation. Annu Rev Microbiol.

[12] Qiu Y-L, Sekiguchi Y, Imachi H, Kamagata Y, Tseng I-C, Cheng S-S (2004). Identification and isolation of anaerobic, syntrophic phthalate isomer-degrading microbes from methanogenic sludges treating wastewater from terephthalate manufacturing. Appl Environ Microbiol.

[13] Boone D, Bryant M (1980). Propionate-degrading bacterium, *Syntrophobacter wolinii* sp. nov., gen. nov., from methanogenic ecosystems. Appl Environ Microbiol.

[14] McInerney MJ, Bryant MP, Hespell RB, Costerton JW (1981). *Syntrophomonas wolfei* gen. nov. sp. nov., an anaerobic, syntrophic, fatty acid-oxidizing bacterium. Appl Environ Microbiol.

[15] Jackson BE, Bhupathiraju VK, Tanner RS, Woese CR, McInerney MJ (1999). *Syntrophus aciditrophicus* sp. nov., a new anaerobic bacterium that degrades fatty acids and benzoate in syntrophic association with hydrogen-using microorganisms. Arch Microbiol.

[16] Mountfort D, Brulla W, Krumholz L, Bryant M (1984). *Syntrophus buswellii* gen. nov., sp. nov.: a benzoate catabolizer from methanogenic ecosystems. Int J Syst Bacteriol.

[17] Iino T, Mori K, Suzuki K (2010). *Methanospirillum lacunae* sp. nov., a methaneproducing archaeon isolated from a puddly soil, and emended descriptions of the genus *Methanospirillum* and *Methanospirillum hungatei*. Int J Syst Evol Microbiol.

[18] Wright AD, Pimm C (2003). Improved strategy for resumptive identification of methanogens using 16S riboprinting. J Microbiol Meth.

[19] Zeikus JG, Bowen VG (1975). Fine structure of *Methanosprillum hungatii*. J Bacteriol.

[20] Kushwaha SC, Kates M, Sprott GD, Smith IC (1981). Novel polar lipids from the methanogen *Methanospirillum hungatei* GP1. Biochim Biophys Acta.

[21] Pagani I, Liolios K, Jansson J, Chen IMA, Smirnova T, Nosrat B (2012). The Genomes OnLine Database (GOLD) v.4: Status of genomic and metagenomic projects and their associated metadata. Nucleic Acids Res.

[22] DOE Joint Genome Institute [http://www.jgi.doe.gov]. Accessed 4 January 2016.

[23] Phred/Phrap/Consed software package [http://www.phrap.com]. Accessed 4 January 2016.

[24] Ewing B, Green P (1998). Base-calling of automated sequencer traces using phred. II. Error probabilities. Genome Res.

[25] Gordon D, Abajian C, Green P (1998). Consed: a graphical tool for sequence finishing. Genome Res.

[26] Hyatt D, Chen GL, Locascio PF, Land ML, Larimer FW, Hauser LJ (2010). Prodigal: prokaryotic gene recognition and translation initiation site identification. BMC Bioinformatics.

[27] Pati A, Ivanova NN, Mikhailova N, Ovchinnikova G, Hooper SD, Lykidis A (2010). GenePRIMP: a gene prediction improvement pipeline for prokaryotic genomes. Nat Methods.

[28] GenePRIMP [http://geneprimp.jgi-psf.org]. Accessed 4 January 2016.

[29] Markowitz VM, Ivanova NN, Chen IMA, Chu K, Kyrpides NC (2009). IMG ER: a system for microbial genome annotation expert review and curation. Bioinformatics.

[30] IMG-ER [http://img.jgi.doe.gov/er]. Accessed 4 January 2016.

[31] TransportDB [http://www.membranetransport.org/]. Accessed 4 January 2016.

[32] TCDB [http://www.tcdb.org/]. Accessed 4 January 2016.

[33] TBD [http://www.transcriptionfactor.org]. Accessed 4 January 2016.

[34] Welte C, Deppenmeier U (1837). Bioenergetics and anaerobic respiratory chains of aceticlastic methanogens. Biochim Biophys Acta.

[35] Rohlin L, Gunsalus RP (2010). Carbon-dependent control of electron transfer and central carbon pathway genes for methane biosynthesis in the Archaean, *Methanosarcina acetivorans* strain C2A. BMC Microbiol.

[36] Jarrell KF, Albers SV (2012). The archaellum: an old motility structure with a new name. Trends Microbiol.

[37] Thomas NA, Bardy SL, Jarrell KF (2001). The archaeal flagellum: a different kind of prokaryotic motility structure. FEMS Microbiol Rev.

[38] Migas J, Anderson KL, Cruden DL, Markovetz AJ (1989). Chemotaxis in *Methanospirillum hungatei*. Appl Environ Microbiol.

[39] Esquivel RN, Xu R, Pohlschroder M (2013). Novel archaeal adhesion pilins with a conserved N terminus. J Bacteriol.

[40] Plugge CM, Henstra AM, Worm P, Swarts DC, Paulitsch-Fuchs AH, Scholten JC (2012). Complete genome sequence of *Syntrophobacter fumaroxidans* strain (MPOB^T^). Stand Genomic Sci.

[41] Sieber JR, Sims DR, Han C, Kim E, Lykidis A, Lapidus AL (2010). The genome of *Syntrophomonas wolfei*: new insights into syntrophic metabolism and biohydrogen production. Environ Microbiol.

[42] Deppenmeier U, Johann A, Hartsch T, Merkl R, Schmitz RA, Martinez-Arias R (2002). The genome of *Methanosarcina mazei:* evidence for lateral gene transfer between Bacteria and *Archaea*. J Mol Microbiol Biotech.

[43] Tamura K, Nei M (1993). Estimation of the number of nucleotide substitutions in the control region of mitochondrial DNA in humans and chimpanzees. Molec Biol Evol.

[44] Tamura K, Stecher G, Peterson D, Filipski A, Kumar S (2013). MEGA6: molecular evolutionary genetics analysis version 6.0. Molec Biol Evol.

[45] Field D, Garrity G, Gray T, Morrison N, Selengut J, Sterk P (2008). Towards a richer description of our complete collection of genomes and metagenomes “Minimum Information about a Genome Sequence” (MIGS) specification. Nat Biotechnol.

[46] Field D, Amaral-Zettler L, Cochrane G, Cole J, Dawyndt P, Garrity GM (2011). The genomic standards consortium. PLoS Biol.

[47] Garrity GM. Names for Life Browser Tool takes expertise out of the database and puts it right in the browser. Microbiol Today 2010, 37:9.

[48] Woese CR, Kandler O, Wheelis ML (1990). Towards a natural system of organisms: proposal for the domains *Archaea*, Bacteria, and Eucarya. Proc Natl Acad Sci USA.

[49] Garrity GM, Holt JG, Garrity GM, Boone DR, Castenholz RW (2001). Phylum AII. *Euryarchaeota* phy. nov. Bergey’s Manual of Systematic Bacteriology.

[50] Garrity GM, Bell JA, Lilburn T, Brenner DJ, Kreig NR, Staley JT (2005). The revised road map to the manual. Bergey’s Manual of Systematic Bacteriology.

[51] Balch WE, Fox GE, Magrum LJ, Woese CR, Wolfe RS (1979). Methanogens: reevaluation of a unique biological group. Microbiol Rev.

[52] Ashburner M, Ball CA, Blake JA, Botstein D, Butler H, Cherry JM (2000). Gene ontology: tool for the unification of biology. The gene ontology consortium. Nat Genet.

