# Supplementary figures and images for: Complete genome sequence of Methanospirillum hungatei type strain JF1
Source: Stand Genomic Sci. 2016 Jan 6;11:2. doi: 10.1186/s40793-015-0124-8 (PMC4704411; doi:10.1186/s40793-015-0124-8)

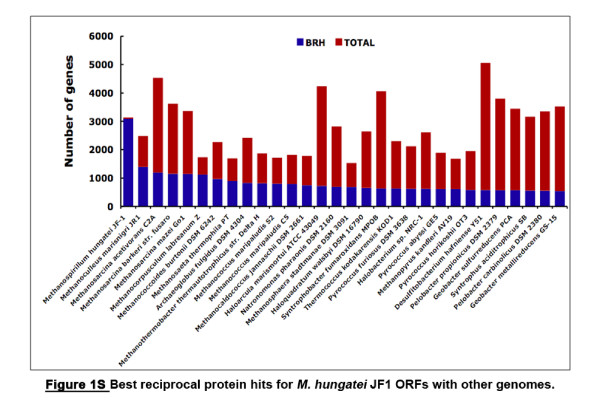

Supplement: Additional file 1: Figure S1. — Best reciprocal protein hits for M. hungatei JF1 ORFs with other genomes. (DOCX 89 kb) [file 40793_2015_124_MOESM1_ESM.docx]

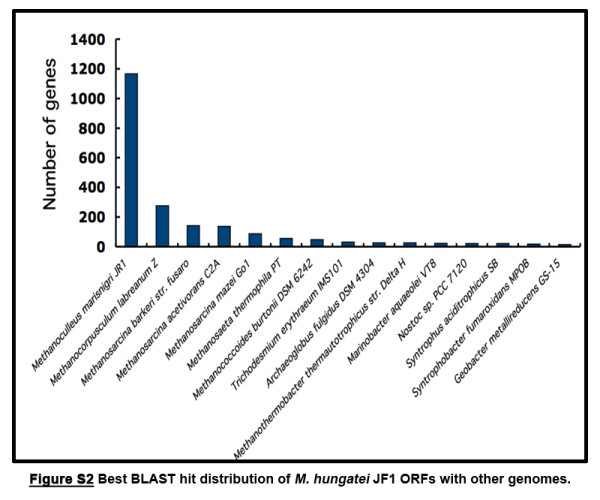

Supplement: Additional file 2: Figure S2. — Best Blast hit distribution of M. hungatei JF1 ORFs with other genomes. (DOCX 62 kb) [file 40793_2015_124_MOESM2_ESM.docx]
